# Real-world clinical effectiveness of trimethoprim–sulfamethoxazole for primary prophylaxis of pneumocystis pneumonia in non-hodgkin lymphoma patients treated with rituximab

**DOI:** 10.1371/journal.pone.0344273

**Published:** 2026-03-03

**Authors:** Patcharaporn Charoenrit, Pimjai Niparuck, Porpon Rotjanapan

**Affiliations:** 1 Department of Medicine, Faculty of Medicine Ramathibodi Hospital, Mahidol University, Bangkok, Thailand; 2 Division of Hematology, Department of Medicine, Faculty of Medicine Ramathibodi Hospital, Mahidol University, Bangkok, Thailand; 3 Division of Infectious Diseases, Department of Medicine, Faculty of Medicine Ramathibodi Hospital, Mahidol University, Bangkok, Thailand; The kids Research Institute Australia, AUSTRALIA

## Abstract

There are no definitive clinical practice guidelines regarding the necessity and dosage of trimethoprim–sulfamethoxazole (TMP/SMX) prophylaxis for *Pneumocystis jirovecii* pneumonia (PJP) in individuals undergoing rituximab therapy. This retrospective study evaluated the effectiveness and safety of various TMP–SMX prophylactic dosing regimens over a 1-year period in 690 patients with non-Hodgkin lymphoma treated with rituximab at a university hospital in Thailand from 2013 to 2022. Out of these patients, 622 (90.1%) received TMP/SMX, with a mean duration of prophylaxis of 265.7 days (SD 85.66). The overall incidence of PJP was 1% (7 patients), which was significantly higher in the non-prophylaxis group (5.8%, 4 patients) compared to the prophylaxis group (0.6%, 3 patients). No cases of PJP occurred among those receiving standard prophylaxis or a single-strength tablet every other day, three times a week. However, instances in the prophylaxis cohort were reported in patients who took two single-strength tablets twice daily, twice a week. Prophylaxis resulted in a significant reduction in the one-year incidence of PJP, with a hazard ratio of 0.105 (95% CI: 0.023–0.469). Mild adverse reactions were noted in 3.05% of patients, all of whom recovered. These findings suggest that TMP/SMX prophylaxis was associated with a lower incidence of PJP and was well tolerated. Future studies should explore optimal dosing strategies while considering patient selection bias and concurrent immunosuppressive therapy.

## Introduction

*Pneumocystis jirovecii* is a fungal pathogen that causes opportunistic infections, particularly pneumocystis pneumonia (PJP), in individuals with compromised immune systems. Historically, these infections were most commonly seen in people with human immunodeficiency virus (HIV) infection, particularly in the context of acquired immunodeficiency syndrome (AIDS). However, advances in antiretroviral therapy and primary prophylactic strategies have significantly reduced the incidence of PJP. One such strategy involves administering trimethoprim–sulfamethoxazole (TMP/SMX) at a single dose of 80/400 mg once or twice daily for patients with a CD4^+^ T-cell count below 200 cells/mm^3^ [1,2]. The widespread availability of this intervention led to a dramatic drop in the annual incidence of PJP among AIDS patients in San Francisco from 95 to 8.4 cases per 1,000 individuals between 1993 and 2008 [3,4].

Despite this progress, the incidence of PJP has been increasing among patients not infected with HIV (non-HIV patients), particularly those receiving immunosuppressive drugs, corticosteroids, hematologic cancer treatments, or bone marrow transplants and those managing rheumatic diseases. In the absence of prophylaxis, the risk of PJP is significantly elevated in patients with lymphoma, particularly in those with pediatric acute lymphoblastic leukemia (22%–45%) and diffuse large B-cell lymphoma (2%–8%). This risk is also notable among organ transplant recipients (5%–15%) and individuals with rheumatic diseases (2%–4%) [5–11]. Patients in these categories frequently present with severe clinical symptoms and experience high mortality rates, ranging from 30% to 60% [12–14]. A study conducted in 2006 at our institution, Ramathibodi Hospital in Bangkok, Thailand, also found that the incidence of PJP among patients with connective tissue diseases undergoing immunosuppressive therapy was 7.6% [15].

Risk factors contributing to the increased incidence of PJP include the long-term use of corticosteroids at doses equivalent to >20 mg/day for at least 1 month. Certain immunosuppressive agents, such as vincristine, cyclophosphamide, and methotrexate, also increase the risk [16]. Another immunosuppressant, rituximab, a monoclonal antibody that specifically targets the CD20 antigen on the surface of B lymphocytes, has become a popular option for treating hematologic malignancies and autoimmune diseases due to its effectiveness in suppressing excessive antibody production. It is commonly used to treat conditions such as lymphoma, rheumatoid arthritis, and systemic lupus erythematosus. However, despite its therapeutic benefits, rituximab can produce adverse effects that include infusion-related reactions, rash, fever, and an increased risk of infections, including PJP [17]. Previous studies have shown that the incidence of PJP in lymphoma patients treated with rituximab ranged from 3% to 15% among those who did not receive primary prophylaxis [10,11,18–22]. Mortality rates at 30, 60, and 90 days after such infection were reported to be 12.1%, 16.6%, and 21.5%, respectively. Fortunately, the use of TMP/SMX for primary prophylaxis significantly improved 1-year survival rates, increasing from 38% to 73% [22]. PJP infections typically occurred between days 69 and 86 after starting lymphoma treatment, with a mean (SD) duration of primary prophylaxis lasting 153.8 (107.6) days [11,23].

According to the European Conference on Infections in Leukemia (ECIL) guidelines 5 (2016), the recommended options for TMP/SMX administration in acute lymphoblastic leukemia, prednisolone equivalent of > 20 mg/day for 4 weeks, fludarabine/cyclophosphamide/rituximab include one single-strength tablet (TMP/SMX 80/400 mg) per day, one double-strength tablet (TMP/SMX 160/800 mg) per day (Grade AII), or one double-strength tablet three times a week (Grade BII). This prophylactic treatment should be continued for at least 6 months following rituximab therapy. However, it is important to note that these recommendations have not been thoroughly validated in patients with other hematologic malignancies, as available data are primarily from small studies and pre–PCR era research. Therefore, the optimal dosage and duration of treatment for preventing PJP remain unclear [16,24].

As a preliminary analysis, we assessed prescribing practices at multiple academic centers in Thailand, which revealed various TMP/SMX dosing regimens used as primary prophylaxis in patients with non-Hodgkin lymphoma receiving rituximab. These regimens include daily administration of one single-strength tablet once daily, two single-strength tablets three times/week, one single-strength tablet three times/week, or two single-strength tablets twice daily two times/week. The attending physician determines the dosage choice, as there is no standardized protocol in Thai clinical practice.

The primary objective of this study was to evaluate the effectiveness of different dosages of TMP/SMX in preventing pneumocystis infections over a one-year period in patients with non-Hodgkin lymphoma who were receiving rituximab for the first time. The secondary objectives included assessing the incidence of other opportunistic infections that are susceptible to TMP/SMX, such as toxoplasmosis and nocardiosis. Additionally, we aimed to examine the incidence of adverse effects associated with TMP/SMX use in lymphoma patients receiving initial rituximab therapy. Specifically, we tested our null hypotheses, which stated that there would be no significant differences in the rates of opportunistic infections, adverse drug events, and overall clinical outcomes among participants receiving various prophylactic dosing regimens.

## Materials and methods

### Study design and participants

This retrospective cohort study examined patients newly diagnosed with non-Hodgkin lymphoma who received chemotherapy alongside the first dose of rituximab within 28 days of their diagnosis. This study was based on patients diagnosed with non-Hodgkin lymphoma at Ramathibodi Hospital, Bangkok, Thailand, between 1 January, 2013, and 31 December, 2022. The study began on 1 November 2023, and subject data were collected from the study to 30 September, 2024. The study population was divided into three groups:

1. Standard Treatment (ST) Group: Participants in this group received either one tablet of single strength once daily (SS OD) or two tablets of single strength once daily (DS OD) three days of the week.
2. Alternative Treatment (AT) Group: Participants received either a low dose of single strength (SS OD) every other day, three days a week, or two tablets of single strength taken twice a day (DS BID) on two days of the week.
3. No Prophylaxis (NP) Group: Participants in this group did not receive any prophylactic treatment.

The following exclusion criteria were applied: pregnancy; positive HIV status; a history of organ transplantation; confirmed cytomegalovirus (CMV) end-organ disease or evidence of CMV DNAemia within the last 3 months; allergy to TMP/SMX; the need for TMP/SMX treatment due to another medical condition; a history of TMP/SMX-sensitive opportunistic infections within the past 6 months; the receipt of dapsone, pentamidine, or TMP/SMX within the last 3 months; estimated glomerular filtration rate of less than 30 ml/min/1.73 m² (TMP–SMX is primarily excreted via the kidneys, dose adjustment is required in patients with an eGFR < 30 mL/min/1.73 m². To ensure patient safety and minimize confounding related to renal impairment and dose modification, patients with an eGFR < 30 mL/min/1.73 m² were excluded from this study.); alanine transaminase level > 5 times the upper normal limit; a history of opportunistic infections sensitive to TMP/SMX within 28 days following the first rituximab dose; loss to follow-up within 28 days after the initial rituximab dose; and incomplete medical records.

### Exposure to PJP prophylaxis

The treating physicians established the prophylactic regimen, defining both the dosage and duration of treatment. The study categorized dosing regimens according to ECIL-5 guidelines, designating one as the standard treatment group and the others as alternative groups. Prophylaxis was administered when the regimen was prescribed to patients within 28 days of initiating rituximab, specifically on day 1, which is the day of the first rituximab administration. Participants who did not meet these criteria were placed in the NP group.

### PJP diagnosis

Participants who met all of the following three criteria were defined as having *definite PJP*: 1) one of the following symptoms consistent with the disease, namely, fever over 38°C, and acute or subacute respiratory symptoms such as dry cough or shortness of breath; 2) chest radiographic findings that were compatible with the disease, such as ground-glass appearance or interstitial pattern; and 3) detection of *Pneumocystis jirovecii* from sputum or bronchoalveolar lavage fluid using the following methods: Giemsa staining, Wright staining, GMS staining, polymerase chain reaction, or immunofluorescent staining. Meanwhile, those who met criteria 1 and 2 above but not criterion 3 were defined as having *probable PJP* [11].

### Outcomes

The primary outcome of this study was the incidence of PJP from day 29 after enrollment until 1 year later. The secondary outcomes focused on the incidence of other opportunistic infections that are susceptible to TMP/SMX included toxoplasmosis and nocardiosis, as well as any adverse effects related to TMP/SMX during the year following enrollment. All patients were monitored starting from day 29 and followed until the occurrence of PJP or other opportunistic infections, death, loss to follow-up, defined as more than 6 months passing since their last visit, or until 12 months after enrollment.

### Statistical analysis

Data were analyzed using STATA version 18.0. Descriptive statistics are presented as means and standard deviations (mean ± SD) for data with a normal distribution. For non-normally distributed data, percentages and medians with minimum and maximum values are reported. For inferential statistics, we compared baseline characteristics (Table 1) between groups using ANOVA for normally distributed data and the Kruskal-Wallis test for non-normally distributed data. Time-to-event outcomes were analyzed using Cox proportional hazards regression to estimate hazard ratios. Incidence outcomes were summarized using descriptive statistics. A p-value of <0.05 was considered statistically significant.

**Table 1:** Baseline clinical and laboratory characteristics of patients in the standard, the alternative, and the non-prophylaxis groups.

Characteristic	Standard group* (n = 147)	Alternative group** (n = 475)	Non-prophylaxis group (n = 68)	p-value
Age, mean (SD), years	63.67 (13.39)	61.43 (13.86)	61.57 (13.62)	0.218
Female, No. (%)	89 (60.54)	255 (53.68)	36 (52.94)	0.321
Body mass index, mean (SD), kg/m^2^	22.99 (3.8)	23.86 (4.13)	23.20 (4.07)	0.051
Comorbidities, No. (%)				
- Hypertension	60 (41.10)	168 (35.37)	27 (39.71)	0.405
- Diabetic mellitus	33 (22.60)	82 (17.26)	12 (17.65)	0.342
- Chronic kidney disease	9 (6.16)	30 (6.32)	3 (4.41)	0.828
- Dyslipidemia	60 (41.10)	141 (29.68)	14 (20.59)	**0.005**
- Coronary artery disease	5 (3.42)	16 (3.38)	3 (4.41)	0.909
- Prior cerebrovascular accident	5 (3.42)	16 (3.37)	0	0.359
- Others	39 (26.71)	108 (22.74)	10 (14.71)	0.149
Hematologic condition, No. (%)				
- Diffuse large B-cell lymphoma	97 (65.66)	321 (67.58)	47 (69.12)	0.026
- Mantle cell lymphoma	1 (0.68)	18 (3.79)	4 (5.88)	
- Follicular lymphoma	12 (8.16)	67 (14.11)	9 (13.24)	
- Marginal zone lymphoma	17 (11.56)	35 (7.37)	5 (7.35)	
- Others	20 (13.61)	34 (7.16)	3 (4.41)	
Duration on rituximab, median (min-max), days	133 (48-364)	139 (19-349)	128.5 (20-365)	0.267
Accumulative dose of rituximab, median (Min, Max), mg	3,500 (1000, 10400)	3,500 (500, 15400)	3,450 (500, 6000)	0.358
Accumulative dose of rituximab, mean (SD), mg/kg	63 (24.77)	59 (23.89)	56 (19.09)	0.057
Baseline white blood cells, median (min-max), cu/mm^3^	7,685 (1,510−94,790)	7,300 (40-92,110)	7,205 (106−43,630)	0.907
Baseline absolute lymphocytes, median (min-max), cu/mm^3^	1,384.7 (115.8−81,519.4)	1,697.45 (6.8-89,346.7)	1,734.8 (9.54-22,687.6)	0.038
Creatinine, mean (SD), mg/dL	0.82 (0.25)	0.86 (0.27)	0.93 (0.29)	0.025
eGFR, mean (SD), mL/min/1.73 m^2^	83.05 (25.69)	80.88 (27.97)	81.28 (22.93)	0.695
Anti-HBc positivity, No. (%)	57 (38.78)	204 (43.13)	17 (30.91)	0.116
First chemotherapy regimen, No. (%)				
- R-CHOP	57 (38.78)	245 (51.58)	27 (39.71)	0.001
- Mini R-CHOP	13 (8.84)	34 (7.16)	1 (1.47)	
- R-CVP	9 (6.12)	29 (6.11)	3 (4.41)	
- R-DAEPOCH	16 (10.88)	19 (4.00)	2 (2.94)	
- R-HyperCVAD	3 (2.04)	5 (1.05)	0	
- CHOP	36 (24.49)	115 (24.21)	31 (45.59)	
- Others	13 (8.84)	28 (5.89)	4 (5.88)	
Relapse or refractory within 1 year, No. (%)	67 (45.58)	242 (50.95)	11 (16.18)	<0.001

A = adriamycin; C = cyclophosphamide; D = dexamethasone; DA = dose adjusted; E = etoposide; eGFR = estimated glomerular filtration rate; H = doxorubicin; O = vincristine; P = prednisolone; R = rituximab; SD = standard deviation; V = vincristine).

*standard group = received either one tablet of single strength once daily (SS OD) or two tablets of single strength once daily (DS OD), three days of the week.

**alternative group = received low dose—single strength (SS OD) every other day, three days of the week or two tablets of single strength twice a day (DS BID) two days of the week.

### Ethics declaration

The study, conducted in accordance with the amended Declaration of Helsinki. The study was approved by the Ethics Committee of Ramathibodi Hospital, Bangkok, Thailand (approval no. MURA ID 2023/748). Written informed consent for participation was not required for this study per the institutional requirements.

## Results

### Baseline characteristics

**Fig 1:**
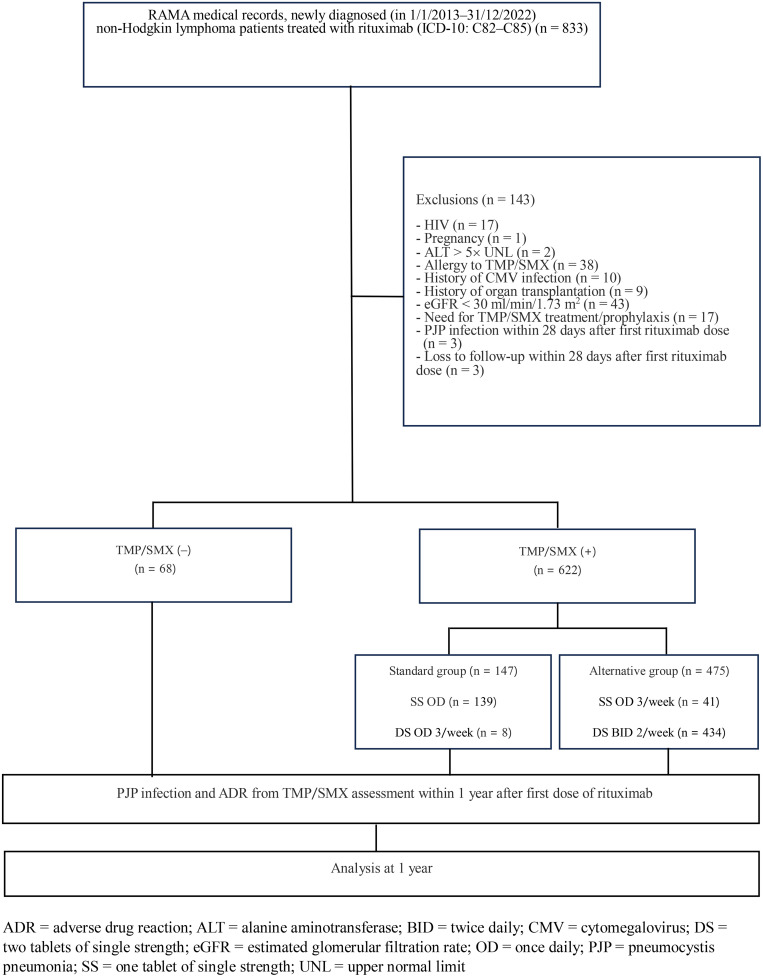
Protocol flow chart.

A search in the Ramathibodi Hospital medical records covering the period from January 1, 2013, to December 31, 2022, identified 833 patients newly diagnosed with non-Hodgkin lymphoma who received chemotherapy combined with rituximab, with or without TMP/SMX for prophylaxis against pneumocystis infection. Among these, 143 patients did not meet the inclusion criteria. Therefore, 690 patients treated with rituximab were analyzed and divided into three groups, namely, 1) ST group (n = 147), 2) AT group (n = 475 cases), and 3) NP group (n = 68 cases), as shown in Fig 1.

The majority of the patients were female (55%). The mean ages for the ST, AT, and NP groups were 63.67 (SD 13.39), 61.43 (+13.86), and 61.57 (+13.62) years, respectively. The most common diagnosis among patients was diffuse large B-cell lymphoma (n = 465/690; 67.4%), followed by follicular lymphoma (n = 88/690; 12.8%) and marginal zone lymphoma (n = 57/690; 8.3%). The baseline characteristics of all participants are presented in Table 1. Among those who received TMP/SMX prophylaxis, the mean time to initiation of the prophylaxis was 1.65 days, with a mean duration of 265.7 days (SD 85.66), starting from the first dose of rituximab. During the study period, 29 patients died after receiving the first dose of rituximab. The prevalence of non-PJP-related deaths, categorized based on available clinical documentation and those without evidence of PJP, was 5.44% (8 out of 147) in the ST group, 3.58% (17 out of 475) in the AT group, and 5.88% (4 out of 68) in the NP group.

### Incidence of opportunistic infections

By the end of the study, seven patients had developed PJP, resulting in an overall incidence rate of 1%. Among these patients, four (5.8%) were in the NP group, which included three definite cases and one probable case. The remaining three patients were in the AT group, with two definite cases and one probable case, yielding an incidence rate of 0.6%. All patients in the AT group received the DS BID regimen on two days of the week. Notably, none of the patients in the ST group or those who received the SS OD regimen, every other day, three days of the week developed PJP.

**Fig 2:**
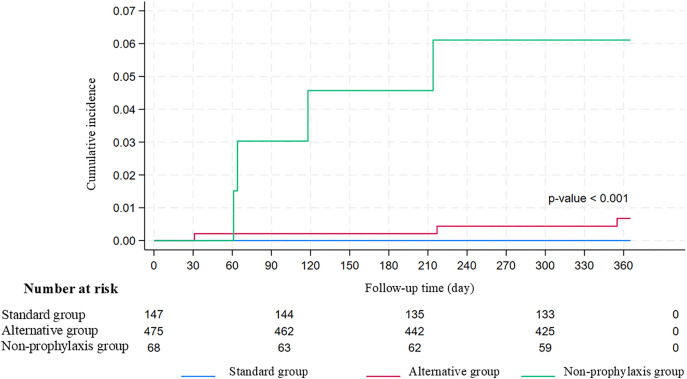
Cumulative incidence of pneumocystis pneumonia in the standard, the alternative, and the non-prophylaxis groups.

The incidence of PJP per 10,000 person-years was as follows: 0.296 across all groups, 0.184 in the AT group, and 1.761 in the NP group (see Fig 2). The median time from the onset of PJP to the first dose of rituximab was 217 days, with a range of 31–355 days. The overall mortality rate associated with PJP was 14.3%, representing one death.

In the DS BID two days of the week group, two out of three cases of PJP were diagnosed after the discontinuation of TMP/SMX, with intervals of 162 and 184 days, respectively. Additionally, one patient had received high-dose corticosteroids due to coronavirus infection (COVID-19) 1 month prior to the PJP diagnosis. Further characteristics of the patients diagnosed with PJP are summarized in Table 2. An intention-to-treat analysis documented four cases of PJP in the NP group and three cases in the TMP/SMX group. Prophylaxis significantly reduced the 1-year incidence of PJP, with a hazard ratio (comparison between AT and NP) of 0.105 (95% confidence interval: 0.023–0.469). No other opportunistic infections sensitive to TMP/SMX were recorded in this study.

**Table 2:** PJP patients in the alternative and the non-prophylaxis groups.

No.	PJP diagnosis	Age (years)	Sex	TMP/SMX regimen	Hematologic condition	Chemo-therapy	Duration from 1^st^ rituximab to PJP (days)	ALC at 1^st^ rituximab (cu/mm^3^)	ALC at PJP diagnosis (cu/mm^3^)	Clinical outcome within 1 year	Clinical data
1.	Prob.	68	F	DS BID 2 times/week	Marginal zone lymphoma	R-B	31	1,130	754	Survived	On prednisolone 10 mg/day, MTX 20 mg/week, leflunomide 20 mg/d, Aza 50 mg/d, HCQ 200 mg/d
2.	D	75	M	DS BID 2 times/week	High-grade B-cell lymphoma	R-CHOP	355	2,415	290	Survived	Diagnosed with Covid 1 month before + high-dose steroid ≥20 mg/d 4 weeks
3	D	47	M	DS BID 2 times/week	Relapsed SLL	R-B	217	23,197	457	Deceased at day 233	
4.	D	67	M	No prophylaxis	DLBCL	R-CHOP	104	417	349	Survived	Dx. chemotherapy induced pneumonitis on prednisolone 60 mg/d 2 weeks then 30 mg/d 1 week then 20 mg/d 1 week then 10 mg/d 1 week then 5 mg/d 1 week
5.	D	73	F	No prophylaxis	DLBCL	R-CHOP	61	1,081	814	Survived	–
6.	D	81	F	No prophylaxis	Marginal zone	R-CVP	214	1,638	449	Survived	On prednisolone 10 mg/day 4 weeks Suspected adrenal insufficiency
7.	Prob.	65	M	No Prophylaxis	DLBCL	CHOP	64	2,779	349	Survived	–

AF = atrial fibrillation; ALC = absolute lymphocyte count; Aza = azathioprine; BID = twice daily; C = cyclophosphamide; D = definite pneumocystis pneumonia; DLBCL = diffuse large B-cell lymphoma; DS = two tablets of single strength; F = female; H = doxorubicin; HCQ = hydroxychloroquine; M = male; mg/d = milligrams/day; MTX = methotrexate; O = vincristine; P = prednisolone; Prob. = probable pneumocystis pneumonia; PJP = pneumocystis pneumonia; R = rituximab; SLL = small lymphocytic lymphoma; TMP/SMX = trimethoprim–sulfamethoxazole; V = vincristine.

### Adverse drug events

Among 622 patients who received TMP/SMX prophylaxis, 19 experienced adverse drug reactions (ADRs) related to TMP/SMX. Overall, 17 of these patients were taking DS BID twice a week, 1 was taking SS OD three times a week, and 1 received the SS OD regimen. The overall incidence rate was 30.55 DRs per 1,000 person-years.

The most commonly reported side effect was leukopenia, which occurred in 10 patients. This was followed by elevated levels of alanine aminotransferase and/or aspartate aminotransferase in 6 patients, and rash in 3 patients. A total of 14 patients (2.25%) who experienced side effects had to discontinue TMP/SMX. The severity of all side effects ranged from grade 1–2, as defined by the Common Terminology Criteria for Adverse Events Version 5.0, and all symptoms resolved after discontinuation of TMP/SMX.

## Discussion

The incidence of PJP is increasing globally among non-HIV patients, particularly those on immunosuppressive therapies, including among lymphoma patients on rituximab and cyclophosphamide, doxorubicin, vincristine, and prednisolone (CHOP-14) chemotherapy without prophylaxis [5,7]. According to research by Elsegeiny et al., B cells play a critical role in supporting T-cell proliferation and differentiation, emphasizing the cooperative function of B and T cells in controlling pneumocystis infections [25]. The fact that rituximab depletes B cells and inactivates/depletes T cells is suggestive of the mechanism by which it increases the risk of PJP. At present, no standardized TMP/SMX prophylactic regimen for non-HIV patients has been established in Thailand, leading to variations in prescribing practices.

A study conducted by Park et al in 2022 involved 3,524 patients (hematologic diseases, rheumatic diseases, and solid organ transplantation) who were treated with rituximab between 2002 and 2018. The patients were divided into two groups: Group 1 included 1,001 patients who received TMP/SMX to prevent primary infection, while Group 2 consisted of 2,523 patients who did not receive this treatment. The results indicated that the incidence of PJP was significantly lower in Group 1. Most participants in the study were administered either an SS OD dose or a DS OD three times a week. The mean duration of TMP/SMX treatment was 153.8 days, with PJP infection occurring on day 86 on average. In both groups, the rate of infection started to plateau approximately 4–6 months after the initiation of treatment [23].

Several studies have compared the use of low-dose TMP/SMX with standard dosing regimens, showing fewer side effects in patients receiving lower doses after 6 months of follow-up [26,27]. For instance, a study in Japan by Utsunomiya et al., published in 2017, evaluated TMP/SMX prophylaxis in patients with systemic rheumatic disease receiving prednisolone at doses of 0.6 mg/kg/day or higher. The participants were divided into three groups based on the TMP/SMX regimen: a single-strength group (TMP/SMX of 80/400 mg daily), half-strength group (40/200 mg daily), and escalation group (started with 8/40 mg daily, increasing incrementally to 40/200 mg daily). The incidence of PJP-free survival at 24 weeks did not differ among these groups. Moreover, the rate of discontinuation due to adverse effects was lower in the half-strength group than in the single-strength group [26].

The current study compared the effectiveness of primary prophylaxis for PJP and evaluated the safety of TMP/SMX at different dosages to determine the optimal regimen for preventing PJP in non-Hodgkin lymphoma patients receiving rituximab. The data revealed that the baseline characteristics, including age, sex, comorbidities, hematologic conditions, and the duration of rituximab administration, were comparable across the three patient groups. The majority of patients were female, diagnosed with DLBCL, and received R-CHOP (rituximab-CHOP) as their initial treatment regimen. Notably, PJP infections were more prevalent among patients over 65 years of age, which was above the average age of the study population.

In our study, the majority of patients had lymphoma, which likely minimized the potential confounding effect of pneumonia caused by PJP related to lymphoma. PJP risk is generally higher in patients with acute lymphoblastic leukemia (ALL), whereas the incidence is relatively low in other lymphoma subtypes [5–11].Specifically, the incidence of PJP in patients treated with rituximab was only 1%, which is considerably lower than previous reports that indicated rates between 2.56% and 2.95% [10,23]. In the NP group, the incidence of PJP was 5.8%, while earlier studies reported rates ranging from 3% to 15% [10,11,18–22]. Meanwhile, the incidence in the AT group was 0.6%, while no PJP cases were observed in the ST group. These findings support existing evidence that primary prophylaxis with TMP/SMX significantly reduces the risk of PJP, emphasizing the protective effect of prophylaxis. Additionally, the calculated HR of 0.105 further underscores the substantial protective benefit of TMP/SMX prophylaxis in patients treated with rituximab, supporting its continued use in this high-risk population.

At the end of the current study, TMP/SMX prophylaxis was associated with a reduced incidence of PJP after 1 year among the study population. There was also no significant difference in this regard between the AT group and the ST group. Notably, no cases of PJP were reported in the low-dose group (SS OD, administered three times a week). In those patients receiving TMP/SMX prophylaxis, all patients diagnosed with PJP received the DS BID twice-weekly regimen. Additionally, one patient with PJP was concurrently using immunosuppressive agents, such as methotrexate, leflunomide, and azathioprine, which may have heightened the risk for PJP. Another patient had been on high doses of corticosteroids (over 20 mg per day) due to COVID-19-related pneumonia one month prior to the PJP diagnosis, further increasing the susceptibility to the infection. The twice-weekly dosing regimen of DS tablets may have led to subtherapeutic drug levels, as sulfamethoxazole and trimethoprim have half-lives of 6–12 hours and 8–10 hours, respectively [28]. To maintain adequate drug levels, a shorter dosing interval of approximately every two days may be more effective.

Regarding adverse side effects, patients receiving DS BID two times a week experienced a higher incidence of hematologic abnormalities. ADRs related to TMP/SMX were generally mild, classified as grade 1–2, with an overall incidence of 3.05%. Additionally, 2.25% of patients had to discontinue TMP/SMX due to these effects. These findings are consistent with previous research, which indicates that TMP/SMX is well-tolerated and has manageable side effects. Considering the significant reduction in the incidence of PJP and associated mortality, our findings suggest that the benefits of prophylaxis outweigh the risks.

The strengths of this study include the use of a database from a large patient population and the stratification of groups based on different TMP/SMX dosage regimens to assess primary prophylaxis efficacy in a real-world setting. However, several limitations of this work should be acknowledged. First, this cohort study was retrospective in nature and conducted at a single center, so caution should be taken when attempting to extrapolate the findings to other contexts. Additionally, patient selection bias may have influenced the results, as the treating physician determined TMP/SMX prophylaxis administration. Second, the distribution of participants was unequal across groups; the NP and ST groups had relatively small sample sizes, which may have led to underestimation of the incidence of PJP, particularly in the ST group, where no PJP cases were observed. In contrast, those receiving the regimen of DS BID two times a week constituted the largest proportion of participants (62.90%), resulting in more PJP cases and TMP/SMX-related adverse effects. Future studies should aim for larger, more balanced sample sizes across groups.

Another limitation of this work is that the follow-up period in this study was relatively short. Among all participants, 46.37% had relapsed or refractory disease, potentially requiring prolonged administration of rituximab and TMP/SMX. The short follow-up period may not have been sufficient to fully assess the long-term outcomes of opportunistic infections. Future studies with extended follow-up beyond 12 months would provide more comprehensive insights. Finally, in addition to evaluating the various chemotherapy regimens among participants, the total dosage of corticosteroids was not specifically examined. This oversight could be significant, as a higher corticosteroid dose may independently increase the risk of PJP. Future studies should include a more thorough assessment of this potential confounding factor to provide a clearer understanding of how pneumocystis infections develop during lymphoma treatment.

## Conclusions

All dosing regimens of TMP/SMX prophylaxis were associated with a reduced incidence of PJP in non-Hodgkin lymphoma patients receiving rituximab in this study. Low-dose regimens provided clinical outcomes comparable to those of higher-dosing regimens in terms of preventing PJP infections, while also having fewer adverse effects at 1-year follow-up. Further study is required to verify the impact of a lower dose of TMP/SMX on opportunistic infections in this population.
